# Isolation of Irkut Virus from a *Murina leucogaster* Bat in China

**DOI:** 10.1371/journal.pntd.0002097

**Published:** 2013-03-07

**Authors:** Ye Liu, Shoufeng Zhang, Jinghui Zhao, Fei Zhang, Rongliang Hu

**Affiliations:** Laboratory of Epidemiology and Key Laboratory of Jilin Province for Zoonosis Prevention and Control, Military Veterinary Research Institute, Academy of Military Medical Sciences, Jingyue Economy Development Zone, Changchun, China; Tulane School of Public Health and Tropical Medicine, United States of America

## Abstract

**Background and objectives:**

Bats are recognized as a major reservoir of lyssaviruses; however, no bat lyssavirus has been isolated in Asia except for Aravan and Khujand virus in Central Asia. All Chinese lyssavirus isolates in previous reports have been of species rabies virus, mainly from dogs. Following at least two recent bat-associated human rabies-like cases in northeast China, we have initiated a study of the prevalence of lyssaviruses in bats in Jilin province and their public health implications. A bat lyssavirus has been isolated and its pathogenicity in mice and genomic alignment have been determined.

**Results:**

We report the first isolation of a bat lyssavirus in China, from the brain of a northeastern bat, *Murina leucogaster*. Its nucleoprotein gene shared 92.4%/98.9% (nucleotide) and 92.2%/98.8% (amino acid) identity with the two known Irkut virus isolates from Russia, and was designated IRKV-THChina12. Following intracranial and intramuscular injection, IRKV-THChina12 produced rabies-like symptoms in adult mice with a short inoculation period and high mortality. Nucleotide sequence analysis showed that IRKV-THChina12 has the same genomic organization as other lyssaviruses and its isolation provides an independent origin for the species IRKV.

**Conclusions:**

We have identified the existence of a bat lyssavirus in a common Chinese bat species. Its high pathogenicity in adult mice suggests that public warnings and medical education regarding bat bites in China should be increased, and that surveillance be extended to provide a better understanding of Irkut virus ecology and its significance for public health.

## Author Summary

The *Lyssavirus* genus presently comprises 12 species and two unapproved species with different antigenic characteristics. *Rabies virus* is detectable worldwide; *Lagos bat virus*, *Mokola virus*, *Duvenhage virus*, *Shimoni bat virus,* and *Ikoma lyssavirus* circulate in Africa; *European bat lyssavirus types 1* and *2*, *Irkut virus*, *West Caucasian bat virus*, and *Bokeloh bat lyssavirus* are found in Europe; and *Australian bat lyssavirus* has been isolated in Australia. Only *Aravan* and *Khujand viruses* have been identified in central Asia. Bats are recognized as the most important reservoirs of lyssaviruses. In China, all lyssavirus isolates in previous reports have been rabies virus, mainly from dogs; none has been from bats. Recently, however, at least two bat-associated human rabies or rabies-like cases have been reported in northeast China. Therefore, we conducted a search for bat lyssaviruses in Jilin province, close to where the first bat-associated human rabies case was recorded. We isolated a bat lyssavirus, identified as an Irkut virus isolate with high pathogenicity in experimental mice. Our findings suggest that public warnings and medical education regarding bat bites in China should be increased, and that surveillance should be extended to provide a better understanding of Irkut virus ecology and its significance for public health.

## Introduction

*Lyssavirus* genus presently comprises 12 species: *rabies virus* (RABV), *Lagos bat virus* (LBV), *Mokola virus* (MOKV), *Duvenhage virus* (DUVV), *European bat lyssavirus types 1* and *2* (EBLV-1 & 2), *Australian bat lyssavirus* (ABLV), *Aravan virus* (ARAV), *Khujand virus* (KHUV), *Irkut virus* (IRKV), *West Caucasian bat virus* (WCBV), and *Shimoni bat virus* (SHIBV) [1]. Additionally, two recent isolates, Ikoma lyssavirus (IKOV) and Bokeloh bat lyssavirus (BBLV), have not yet been assessed taxonomically [2], [3].

Bats are the most important reservoirs of lyssaviruses [1]; however, little is known about bat lyssaviruses in Asia. ARAV and KHUV were isolated in Central Asia [1], and IRKV in eastern Siberia and the Russian Far East [1]. In 2009, a human rabies case was reported in Primorsky Kray, Russian Far East, in a patient bitten by an insectivorous bat. The isolated virus, Ozernoe, was closely related to IRKV [1], [4].

A 1998 survey in Philippines found that 22/231 (9.52%) serum samples from insectivorous bats contained neutralizing antibody (VNA) to ABLV [5]. In Cambodia, 2000-1, 30/187 (16.04%) bat serum samples were found to have VNA against different lyssaviruses [6]. In Thailand, 2002-3, 16/394 (4.06%) serum samples from fruit bats had detectable VNA against ARAV, KHUV, IRKV, or ABLV [7]. Three of 288 (1.04%) bat serum samples collected in Bangladesh during 2003-4 cross-reacted with ARAV and KHUV [8], and in China, 2010, low levels of rabies VNA were demonstrated in 15/685 (2.19%) bat serum samples [9]. However, there has been no report of bat lyssavirus isolation in any of these countries. Although isolation of “rabies” viruses has been reported from Thailand [10] and India [11], these isolates were not characterized or stored for further studies.

In 2002 and 2010, two bat-associated human rabies cases were reported in northeast China, in Tonghua county and Longjing city respectively [12], [13]. One (2002) was reported to the local Jilin Center for Disease Control and Prevention; the other (2010) was not. The victims suffered bat bites on ear and hand respectively. Both cases developed an acute progressive encephalomyelitis which consistent with rabies infection, 30 and 20 days after the bites, and both died 10 days following the first manifestation of disease symptoms. However, the both cases were diagnosed only clinically, without laboratory confirmation. Additionally, according to the information provided by local residents, there was another human death from rabies following a bat bite in Tonghua county in the 1990s.

Since 2002, efforts have been made to obtain human specimens in hospitals and bat specimens in the field. Human specimens have not been obtained, however, since bat-associated human rabies is rare, even though there have been several reports of bat bites [13]–[15]. To investigate more extensively the prevalence of lyssaviruses in bats in Jilin province and their public health implications, therefore, we conducted the field survey described below.

## Materials and Methods

### Ethics statement

All animals experiments described in this paper have been conducted according to the Guideline on the Humane Treatment of Laboratory Animals stipulated by the Ministry of Science and Technology of the People's Republic of China (MOST) [16] and approved by the Animal Welfare Committee of the Military Veterinary Research Institute, Changchun, China. All animals were housed in a climate-controlled laboratory with a 12 h day/12 h night cycle. No human patient derived clinical materials and non-human primates were used in the completion of these studies.

### Sample collection and assay

In April 2012, 261 bats of five species, *Plecotus auritus* (n = 12), *Murina leucogaster* (n = 104), *Myotis daubentonii* (n = 66), *Rhinolophus ferrumequinum* (n = 59), and *Myotis ikonnikovi* (n = 20), were collected in Tonghua county, Jilin province (i.e., where the first bat-associated human rabies case was recorded in 2002) and identified by both morphological traits and amplification and sequencing of the cytochrome B (*cytB*) gene as described previously [17]. This area is also close to Longjing city where the other human case was reported in 2010, and is about 800 km from where a similar human case reported in Russia in 2009 [4]. The bats were captured from stone interstices within an abandoned stone factory using a net with a long handle. All bats were alive and apparently healthy when collected. The animals were humanely euthanized, and their brains were collected and removed for testing by the direct fluorescent antibody (DFA) test [18]. The DFA was performed using LIGHT DIAGNOSTICS Rabies FITC-globulin conjugate (EMD Millipore Corporation, MA, USA) that can detect rabies-related lyssaviruses including rabies virus according to the manufacturer's instructions. Nested reverse transcription-polymerase chain reaction (RT-PCR) was used to obtain the lyssavirus sequences as previously described [18]. PCR products were purified and nucleotide sequencing was performed on both forward and reverse strands of each fragment by Genscript Nanjing Co., China. The sequences were compared with those of previously characterized lyssaviruses using the basic local alignment search tool (BLAST).

### Viruses and cells

Rabies virus BD06 (GenBank #EU549783.1), isolated in our laboratory in 2006 from a rabid dog in China, was maintained in dog brain and could cause ≥80% mortality via i.m. injection in unvaccinated dogs in previous experiments [19].

Mouse neuronal-2a (N2a) cell line was maintained in RPMI1640 medium supplemented with 10% fetal bovine serum, 100 U/mL penicillin G, and 100 µg/mL streptomycin sulfate at 37°C in a 5% CO_2_ humidified incubator.

To compare the pathogenicities of lyssavirus isolate and BD06 at the same dose in mice, both kinds of these viruses were cultivated in N2a cells as described previously [19]. Only two passages in the infected N2a cells were performed to minimize cell adaptation and possible reduction of pathogenicity. The titers of lyssavirus isolate and BD06 in N2a cells were assayed as described previously [19] and were from 10^5.0^–10^5.5^ TCID_50_/mL.

### Animals and virus inoculation

One-day-old suckling and 8-week-old adult Kunming mice were purchased from the Changchun Institute of Biological Products, and respectively used for lyssavirus isolation from the DFA-positive samples and *in vivo* pathogenicity assay. Virus isolation was conducted using the mouse inoculation test (MIT) method as described previously [20].

For pathogenicity, serial 10-fold dilutions of the viruses (second passage in N2a cells) were prepared in PBS/2% horse serum and injected i.m. (the hindlimb skeletal muscle) into 4 groups of 10 adult mice (0.1 mL containing 10^1^–10^4^ TCID_50_; 1 dilution per group).

All the inoculated mice were observed for ∼28 days and the sick and the final survived mice were euthanized by cervical dislocation respectively before their death and at the end of the experimental period. The infected mice were re-checked for the presence of lyssavirus by DFA.

### Viral RNA isolation, primer designs, amplification and sequencing

Preparation of total RNA from bat brain tissue was conducted with TRIzol (Invitrogen Life Technologies, USA) according to the supplier's instructions.

For amplification and analysis of the viral genomes, a total of 9 pairs of primers of overlapping fragments covering the full length of the viral cDNA were designed based on the similar sequences identified using BLAST. Fragments containing the 3′ and 5′ termini of the viral genome were obtained using a 3′ and 5′ Full RACE Kit (TaKaRa Biotechnology Co., Ltd., Dalian, China). The PCR products were purified, and nucleotide sequencing was performed on both forward and reverse strands of each fragment by Genscript Nanjing Co., China.

### Sequence alignment and phylogenetic analysis

Phylogenetic tree was constructed using the Maximum Likelihood method in MEGA 5.10 [21], in which the reliability of the phylogeny groupings was evaluated using bootstrapping with 1000 replicates. The general time-reversible model incorporating invariant sites and a GTR+I+γ_4_ model was favored for all datasets. The percentage identities and similarity scores of the viral protein sequences were calculated using DNAstar Lasergene software. (DNASTAR, Inc., Madison, USA).

Complete genome sequences of each lyssavirus species were used as follows: RABV (16 sequences), LBV (4 sequences), MOKV (2 sequences), DUVV (4 sequences), EBLV-1 (5 sequences), IRKV (3 sequences), EBLV-2 (2 sequences), ABLV, ARAV, KHUV, WCBV, BBLV and SHIBV (1 sequence each).

## Results

### Virus isolation

Of all 261 bat specimens tested, only a single brain sample from an adult male *Murina leucogaster* bat was found positive by DFA. From this sample, a 255-bp sequence was obtained using nested RT-PCR. Using BLAST, its sequence was found to be highly similar (92%/98%) to that of both IRKVs, especially isolate Ozernoe. All the eight inoculated suckling mice with lyssavirus isolate developed neurological signs between days 7–9 post-inoculation, at which point they were euthanized. Each mouse brain sample was DFA positive. During the next intracranial passage, the incubation period was reduced to 3–4 days in suckling mice. All the other bat brain samples were DFA negative.

**Figure 1:**
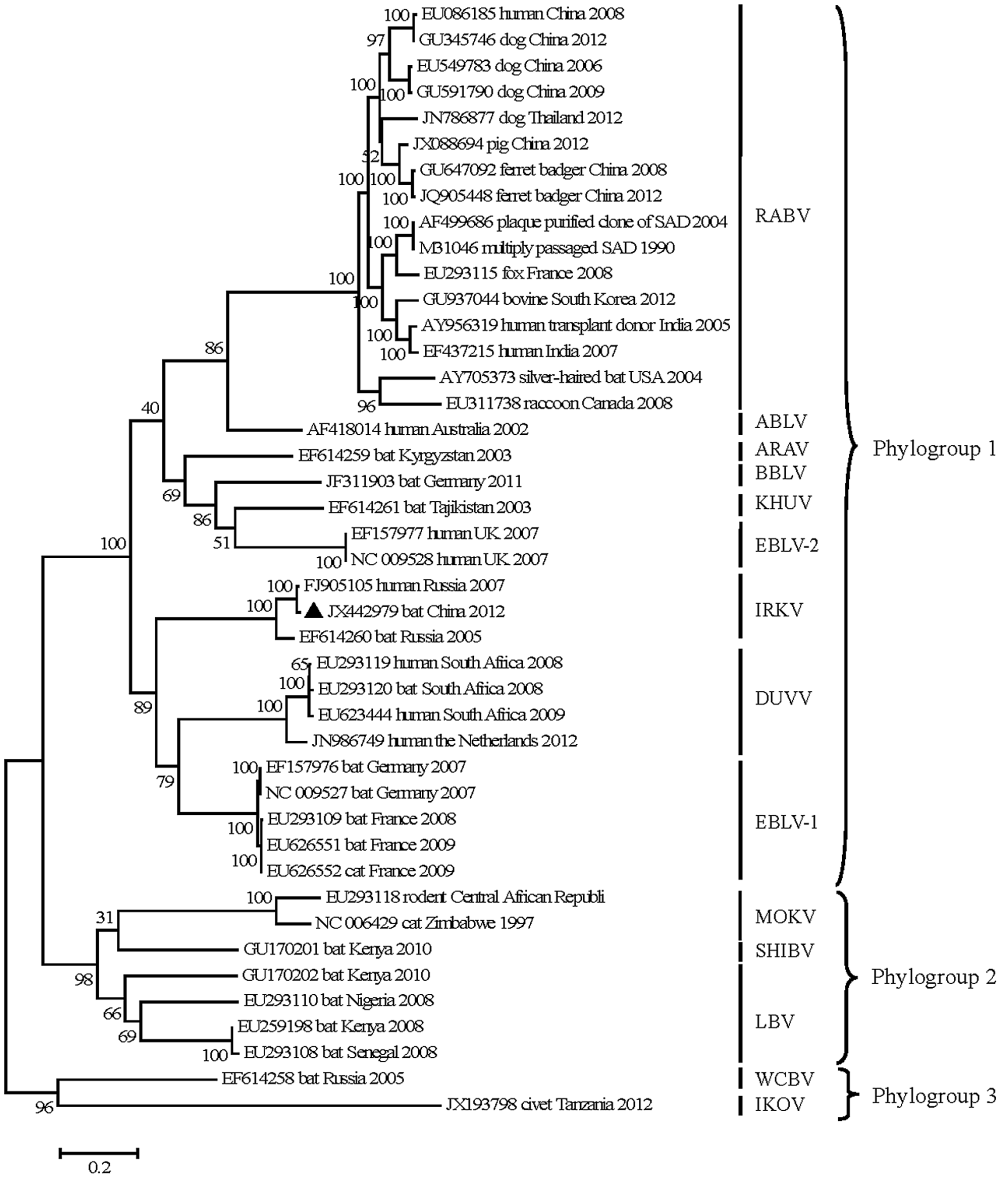
Maximum likelihood of the lyssavirus-rooted phylogenetic tree based on the complete N sequences. The isolate, IRKV-THChina12, is marked using black triangle. Bootstrap values are presented as percentages for key nodes on the tree, and branch lengths are indicated using a scale bar. EBLV-1: European bat lyssavirus-1; DUVV: Duvenhage virus; IRKV: Irkut virus; ARAV: Aravan virus; BBLV: Bokeloh bat lyssavirus; KHUV: Khujand virus; EBLV-2: European bat lyssavirus-2; ABLV: Australian bat lyssavirus; RABV: rabies virus; WCBV: West Caucasian bat virus; SHIBV: Shimoni bat virus; MOKV: Mokola virus; LBV: Lagos bat virus; and IKOV: Ikoma lyssavirus.

As originally proposed by Kuzmin et al., N gene nucleotide identities provided unequivocal separation of all lyssavirus species with an identity threshold of 82% [22]. Alignment analysis based on the full nucleoprotein (N) sequences clearly demonstrated that the isolate clustered with IRKV (Figure 1), sharing 92.4%/98.9% (nucleotide) and 92.2%/98.8% (amino acid) identity (Table 1) with the two available isolates from eastern Siberia and the Russian Far East. The identity within the N sequences for other lyssaviruses (Table 1) ranged from 70.0%–79.2% (nucleotide) and 70.2%–79.1% (amino acid), indicating that the virus is an isolate of the species IRKV [22], [23] and therefore was designated IRKV-THChina12 (“TH” representing Tonghua county and “12”, 2012). The complete genome sequence of IRKV-THChina12 was submitted to GenBank (Accession no. JX442979).

**Table 1:** Comparison of nucleotide and amino acid identity (%) between IRKV-THChina12 and other IRKV isolates.

Genome organization	IRKV isolates	
	Irkut virus (EF614260)	Ozernoe virus (FJ905105)
3′ UTR	98.7	100
N protein	92.4 (92.2)	98.9 (98.8)
N–P UTR	92.1	97.0
P protein	91.3 (91.1)	98.8 (98.6)
P–M UTR	88.0	98.9
M protein	91.9 (91.6)	99.4 (99.0)
M–G UTR	98.2	98.2
G protein	91.0 (90.9)	98.9 (98.7)
G–L UTR	87.4	97.6
L protein	91.1 (91.1)	98.2 (98.2)
5′ UTR	88.6	99.3
Genome	91.2	98.5
G+C content of genome	44.5	44.7

Numbers: nucleotide identity (amino acid identity).

### Pathogenicity of IRKV-THChina12

Following i.m. injection of adult mice at the same doses, rabies-like symptoms were observed 6–10 d post-injection of IRKV-THChina12, whereas the incubation period of BD06 was 8–11 d. All injected adult mice died 24–48 h following appearance of rabies-associated symptoms such as lethargy, ruffled hair, muscle weakness, loss of body weight and progressive paralysis of one or both hind limbs. Table 2 shows that 10/10 adult mice died of IRKV-THChina12 injection at dose 10^4^ TCID_50_, with 7/10 dying from BD06 injection at the same dose. At lower doses (10^2^–10^3^ TCID_50_), some animals survived in every group; however, given 10^2^ TCID_50_ or more, the mortalities in each IRKV-THChina12 group were higher than that in BD06 groups at the same dose.

**Table 2:** Pathogenicity of IRKV-THChina12 and BD06 in adult mice after i.m. injection.

Virus	Deaths per 10 animals at different doses			
	10^1^ TCID_50_	10^2^ TCID_50_	10^3^ TCID_50_	10^4^ TCID_50_
IRKV-THChina12	0	2	4	10
BD06	0	1	2	7

### Genomic organization of IRKV-THChina12

The genome of IRKV-THChina12 was determined to be 11980 nucleotides (nt) in length with a G+C content of 44.72%, similar to the G+C content of 44.49% of Irkut virus and 44.72% of Ozernoe virus (Table 1). The genomic organization was as follows: 70-nt 3′ untranslated region (UTR), 1356-nt nucleoprotein (N), 92-nt N-P UTR, 897-nt phosphoprotein (P), 83-nt P-M UTR, 609-nt matrix protein (M), 214-nt M-G UTR, 1575-nt glycoprotein (G), 569-nt G-L UTR, 6384-nt large protein (L) and 131-nt 5′ UTR. The coding (CDS) and UTR sequences of IRKV-THChina12 were located at the same positions as in other IRKV isolates, with no variation in length.

Table 1 provides an intragenotypic comparison of the five structural proteins of the current and previously published IRKV sequences. Interestingly, the percent identity orders were different between IRKV-THChina12/Irkut virus (N>M>P>L>G) and IRKV-THChina12/Ozernoe virus (M>N>G>P>L), and did not follow the general order (N>L>M>G>P) [24]. However, N and M had very similar percent identities, as did G, P and L, for both nucleotide and amino acid comparisons.

### Phylogenetic analysis

As shown in Figure 1, all lyssaviruses studied can be separated into three major branches previously defined as different “phylogroups” [25]. Although IRKV, ARAV, BBLV, KHUV DUVV, WCBV, EBLV-1 and EBLV-2 within phylogroup 1 have all been found in insectivorous bats widely distributed in Eurasia, IRKV, ARAV, KHUV and WCBV have also been isolated in countries neighbouring China. IRKV-THChina12 was closely related to EBLV-1 and DUVV as well as the other IRKVs, somewhat divergent from ARAV and KHUV, and most divergent from WCBV and IKOV within phylogroup 3. These data are consistent with that of a previous study [22]. The tree summarizing an analysis of the whole N gene (Figure 1) showed that IRKV-THChina12 clustered with isolate Ozernoe from the China-Russian border and with original isolate Irkut virus.

IRKV-THChina12 and the two other IRKV isolates all provide independent origins for the species IRKV. However, according to the branch length, IRKV-THChina12 has undergone more changes since the three isolates diverged from their common ancestor; i.e., the evolution rate in the lineage leading up to IRKV-THChina12 is greater than that leading up to the isolate Ozernoe. It has been previously suggested that complete genomes provide the best tools for genotyping [26]. In this study, similar conclusions were drawn from a full genome analysis (data not shown).

## Discussion

The identification of IRKV-THChina12 represents the first isolation of a bat lyssavirus in China. Although it was found in only a single sample (1/104) from *Murina leucogaster* bats, other considerations suggest that this bat species should receive special attention concerning its association with IRKV. The greater tube-nosed bat (*Murina leucogaster*) is a non-migratory species, distributed throughout India, Mongolia, Siberia and the Far East of Russia, China, Korea, and Japan [26]. The original IRKV isolate was obtained from this species [1], as was our isolate. Potentially, while the circulation patterns of IRKV in all bat populations require more extensive surveillance since only a single isolate was obtained in this study, it is likely that the greater tube-nosed bat (*Murina leucogaster*) is the principal vector of the virus. IRKV may circulate in bats of this species across all this vast territory.

The overall genomic organization of the isolate IRKV-THChina12 is similar to those of IRKVs and other lyssaviruses [27], with minor variations. Although complete sequences of both IRKV genomes have been available for several years, neither has been used to estimate the evolutionary relationship among lyssaviruses. Differences in rates of evolution are common and can be due to many factors, such as different mutation rates, population sizes and selective forces. Therefore, it remains to be determined whether emergence of the IRKV-THChina12 occurred as a result of adaptation of Ozernoe or another IRKV to Chinese bats.

At the genus level, the N gene is the most conserved protein-coding region, followed in order by L>M>G>P with minor variations [28]–[30]. Within the species IRKV, the percent identity orders differ among isolates, but the similarity scores for amino acid compared with nucleotide alignments suggest that the majority of the changes at the nucleotide level are synonymous. High conservation of the N, G, P and L proteins of IRKV has been noted in previous reports [22], [31]. However, in comparison with isolate Ozernoe, the M gene of IRKV-THChina12 was more conserved than the N, P, G and L genes.

In previous studies, the pathogenicity of strain BD06 was the highest of all rabies virus isolates stored in our laboratory, including Chinese ferret badger isolate [19], Shaanxi-HZ-6 (GU591790) and GN07 (EU828653). In mice, the pathogenicity of IRKV-THChina12 was characterized by a shorter inoculation period and higher mortality than that of BD06, indicating that it poses a potential risk to humans and animals. As commercial rabies biologics have been demonstrated to provide only a partial cross-protection against IRKV [32], the need for development and evaluation of new biologics requires further assessment to ensure adequate protection against IRKV in China.

## Supporting Information

Figure S1 Tonghua county and surrounding regions in Russia and China. Black dots indicate the location of IRKV isolates and/or IRKV-associated human rabies cases within China and Russia. The red dot identifies where IRKV-THChina12 virus was isolated.
(DOC)

Table S1 Primers used for amplification and sequencing of the IRKV-THChina12 genome.
(DOC)

Table S2 Lyssavirus sequences used in the present study.
(DOC)
