# Supplementary material for: Isolation of Irkut Virus from a Murina leucogaster Bat in China
Source: PLoS Negl Trop Dis. 2013 Mar 7;7(3):e2097. doi: 10.1371/journal.pntd.0002097 (PMC3591329; doi:10.1371/journal.pntd.0002097)
Supplement: Figure S1 — Tonghua county and surrounding regions in Russia and China. Black dots indicate the location of IRKV isolates and/or IRKV-associated human rabies cases within China and Russia. The red dot identifies where IRKV-THChina12 virus was isolated. (DOC) [file pntd.0002097.s001.doc]

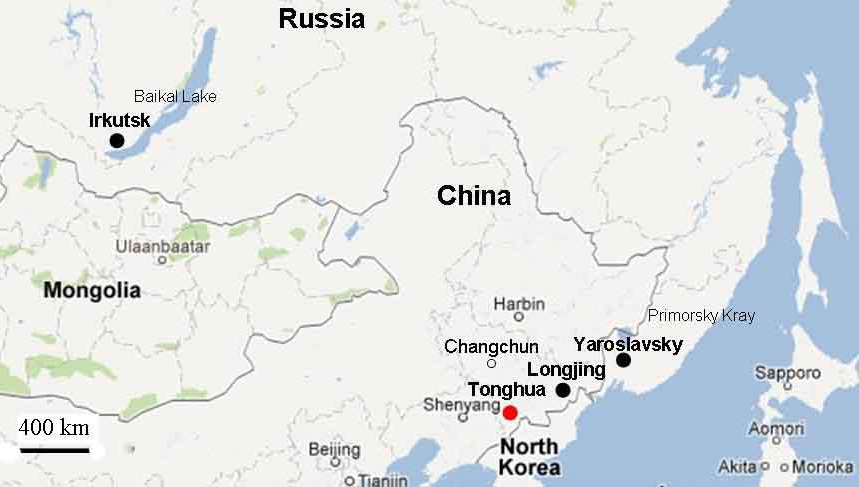


Figure S1. Tonghua county and surrounding regions in Russia and China.

Black dots indicate the location of IRKV isolates and/or IRKV-associated human rabies cases within China and Russia. The red dot identifies where IRKV-THChina12 virus was isolated.
