# Supplementary material for: Isolation of Irkut Virus from a Murina leucogaster Bat in China
Source: PLoS Negl Trop Dis. 2013 Mar 7;7(3):e2097. doi: 10.1371/journal.pntd.0002097 (PMC3591329; doi:10.1371/journal.pntd.0002097)
Supplement: Table S1 — Primers used for amplification and sequencing of the IRKV-THChina12 genome. (DOC) [file pntd.0002097.s002.doc]

Table S1 Primers used for amplification and sequencing of the IRKV-THChina12 genome

| Name | Sequence (5’-3’) | Genome position | Sense |
| --- | --- | --- | --- |
| F1F | 5’-GGTAGACATGTTCATCTGTAG-3’ | 27-47 | Positive |
| F1R | 5’-ATTGGGTCTAGCCTGATGGT-3’ | 1359-1378 | Negative |
| F2F | 5’-ACTAGAAGCCCTGAAGCAGT-3’ | 1265-1284 | Positive |
| F2R | 5’-ATGACTTCAGGATGTGTCGC-3’ | 2714-2733 | Negative |
| F3F | 5’-GATGACGACGATCTGTGGCT-3’ | 2577-2596 | Positive |
| F3R | 5’-ATCCACACAGTCTGCCGTTC-3’ | 3984-4003 | Negative |
| F4F | 5’-GAGAAACTCAGCATGTCCTG-3’ | 3925-3944 | Positive |
| F4R | 5’-CCATTCGGTGGAGGTCTAAG-3’ | 5302-5321 | Negative |
| F5F | 5’-TTCCAGTGATCCGAACTGCG-3’ | 5160-5179 | Positive |
| F5R | 5’-TAGGTGAAGACGAGGTCATG-3’ | 6540-6559 | Negative |
| F6F | 5’-CTCAATTCATCCGAGACAAG-3’ | 6445-6464 | Positive |
| F6R | 5’-TAACTGCACATAGTCTCGTC-3’ | 7803-7822 | Negative |
| F7F | 5’-CCAGGTGTTGTGTCCGACGT-3’ | 7655-7674 | Positive |
| F7R | 5’-GTTCGCTTGAATACAGGAGC-3’ | 9153-9172 | Negative |
| F8F | 5’-ACTGGTTCATCACCAGAGAT-3’ | 9064-9083 | Positive |
| F8R | 5’-CCAACAACCAAGCAGAGTGT-3’ | 10557-10576 | Negative |
| F9F | 5’-AGATGGATGTCCGAGCACT-3’ | 10428-10447 | Positive |
| F9R | 5’-GAATGTAGAAATGCTGGCTG-3’ | 11920-11939 | Negative |
