# Supplementary material for: Isolation of Irkut Virus from a Murina leucogaster Bat in China
Source: PLoS Negl Trop Dis. 2013 Mar 7;7(3):e2097. doi: 10.1371/journal.pntd.0002097 (PMC3591329; doi:10.1371/journal.pntd.0002097)
Supplement: Table S2 — Lyssavirus sequences used in the present study. (DOC) [file pntd.0002097.s003.doc]

Table S2 Lyssavirus sequences used in the present study

| species | Strain | Isolation or origin | Year | GenBank no. |
| --- | --- | --- | --- | --- |
| RABV | 9147FRA | fox, France | 2008 | EU293115 |
|  | JX12-67 | ferret badger, China | 2012 | JQ950448 |
|  | Shaanxi-HZ-6 | dog, China | 2009 | GU591790 |
|  | GD-SH-01 | pig, China | 2012 | JX088694 |
|  | CQ92 | dog, China | 2012 | GU345746 |
|  | 02050CHI | human, China | 2008 | EU086185 |
|  | KRVB0903 | bovine, South Korea | 2012 | GU937044 |
|  | QS-05 | dog, Thailand | 2012 | JN786877 |
|  | NNV-RAB-H | human, India | 2007 | EF437215 |
|  | SAD B-19 | multiply passaged SAD | 1990 | M31046 |
|  | SRV_9_ | plaque purified clone of SAD | 2004 | AF499686 |
|  | BD06 | dog, China | 2008 | EU549783 |
|  | SHBRV-18 | silver-haired bat, USA | 2004 | AY705373 |
|  | Hum-Trans-IND | human transplant donor, India | 2005 | AY956319 |
|  | JX08-45 | ferret badger, China | 2008 | GU647092 |
|  | RRV ON-99-2 | raccoon, Canada | 2008 | EU311738 |
| LBV | KE131 | bat, Kenya | 2008 | EU259198 |
|  | KE576 | bat, Kenya | 2010 | GU170202 |
|  | 0406SEN | bat, Senegal | 2008 | EU293108 |
|  | 8619NGA | bat, Nigeria | 2008 | EU293110 |
| MOKV | Mokola virus | cat, Zimbabwe | 1997 | NC_006429 |
|  | 86101RCA | rodent, Central African Republic | 2008 | EU293118 |
| DUVV | NL07 | human, the Netherlands | 2012 | JN986749 |
|  | 86132SA | human, South Africa | 2008 | EU293119 |
|  | 94286SA | bat, South Africa | 2008 | EU293120 |
|  | DUVVSA06 | human, South Africa | 2009 | EU623444 |
| EBLV-1 | European bat lyssavirus 1 | bat, Germany | 2008 | NC_009527 |
|  | 03002FRA | bat, France | 2008 | EU293109 |
|  | 08120FRA | bat, France | 2009 | EU626551 |
|  | 07240FRA | cat, France | 2009 | EU626552 |
|  | RV9 | bat, Germany | 2007 | EF157976 |
| EBLV-2 | European bat lyssavirus 2 | human, UK | 2007 | NC_009528 |
|  | RV1333 | human, UK | 2007 | EF157977 |
| ABLV | Australian bat lyssavirus | human, Australia | 2002 | AF418014 |
| ARAV | Aravan virus | bat, Kyrgyzstan | 2003 | EF614259 |
| IRKV | Irkut virus | bat, Russia | 2005 | EF614260 |
|  | Ozernoe virus | human, Russia | 2007 | FJ905105 |
|  | IRKV-THChina12 | bat, China | 2012 | JX442979 |
| KHUV | Khujand virus | bat, Tajikistan | 2003 | EF614261 |
| WCBV | West Caucasian bat virus | bat, Russia | 2005 | EF614258 |
| SHIBV | Shimoni bat virus | bat, Kenya | 2010 | GU170201 |
| BBLV* | Bokeloh bat lyssavirus | bat, Germany | 2011 | JF311903 |
| IKOV* | Ikoma lyssavirus | civet, Tanzania | 2012 | JX193798 |

*Unapproved as species.
